# Case Report: A rare pediatric case of secretory carcinoma of the parotid gland with high-grade components misdiagnosed as pleomorphic adenoma

**DOI:** 10.3389/fonc.2026.1797821

**Published:** 2026-05-29

**Authors:** Li Xu, Huihua He, Honglin Yan, Xiaoli Tian, Yongfei Tang, Jingping Yuan

**Affiliations:** Department of Pathology, Renmin Hospital of Wuhan University, Wuhan, China

**Keywords:** high-grade, parotid gland, pediatric case, pleomorphic adenoma, secretory carcinoma

## Abstract

**Background:**

Secretory carcinoma (SC) of the parotid gland is an extremely rare malignant neoplasm in childhood. Given its close clinical and imaging similarities to pleomorphic adenoma, as well as the high potential for diagnostic inaccuracies in fine-needle aspiration cytology (FNAC), preoperative diagnosis is often clinically challenging. Pediatric patients with salivary gland tumors are frequently misdiagnosed, which may lead to unfavorable clinical consequences.

**Case presentation:**

A 12-year-old male presented with an asymptomatic left parotid mass of 6 months’ duration. Preoperative fine-needle aspiration cytology was suggestive of pleomorphic adenoma. Magnetic resonance imaging (MRI) demonstrated a well-circumscribed nodule measuring 2.5 cm in maximum diameter. Intraoperative frozen section favored a low-grade malignant salivary gland tumor. The patient underwent superficial parotidectomy with functional neck dissection (levels I-III). Histopathological examination revealed the following characteristic features of the tumor: small, monomorphic epithelial cells with eosinophilic cytoplasm abundant in secretory granules; frequent microcystic or follicular architectures containing secretory material within the cystic lumina. Additionally, localized high-grade morphological features were identified, including solid areas accounting for more than 30%, nuclear enlargement with prominent nucleoli, discernible mitotic figures, and tumor giant cells. Periodic acid-Schiff (PAS) staining revealed positive secretions rich in glycogen and mucin. Immunohistochemically, the tumor cells were positive for CK19, S100, SOX10, and Mammaglobin. No perineural or lymphovascular invasion was identified. Fluorescence *in situ* hybridization (FISH) confirmed the presence of an *ETV6::NTRK3* gene fusion. Postoperative recovery was uneventful. Multidisciplinary team (MDT) discussion concluded that adjuvant therapy was unnecessary. At 29 months of follow-up, the patient remained disease-free with no evidence of recurrence, preserved facial nerve function, and no radiological signs of residual or recurrent disease.

**Conclusion:**

We reported a rare case of non-metastatic parotid SC with high-grade components in a child, which was successfully managed by surgical resection alone. Preoperative clinical manifestations, imaging findings, and FNAC collectively suggested a diagnosis of pleomorphic adenoma. Intraoperative frozen section examination indicated a pathological diagnosis of low-grade malignant salivary gland tumor. Thus, comprehensive histopathological and molecular analyses are essential, as the morphological features of this tumor may be deceptively bland.

## Introduction

Malignant salivary gland tumors constitute less than 5% of all pediatric head and neck neoplasms (1). These tumors exhibit a slight female predominance (57.4%) (2). Among these entities, secretory carcinoma (SC), initially designated as mammary analogue secretory carcinoma owing to its striking histological resemblance to secretory breast carcinoma, is an extremely rare malignancy defined by a recurrent *ETV6::NTRK3* gene fusion, which is associated with the translocation t (12;15) (p13; q25). It accounts for fewer than 1% of all salivary gland cancers in the pediatric population (3). Since its recognition as a distinct entity in 2010, only a limited number of cases involving patients younger than 16 years of age have been documented in the medical literature (3–5). Children exhibiting SC with high-grade components are exceedingly rare. The extreme rarity of this tumor in children poses significant diagnostic challenges to pathologists, who may have limited experience with its morphological and immunohistochemical profiles. Furthermore, no standardized, pediatric-specific clinical guidelines for the management of SC are currently available. Critical questions concerning the optimal extent of surgical resection, the indications for elective neck dissection in clinically node-negative cases, and the role of adjuvant radiotherapy remain contentious. Additionally, the potential application of targeted *NTRK* inhibitor therapy in pediatric patients with advanced or recurrent disease represents an emerging yet poorly delineated area of clinical decision-making. Given this context, we detail the case of a 12-year-old boy with localized parotid secretory carcinoma who was successfully treated with radical surgery alone.

## Case presentation

A previously healthy 12-year-old boy incidentally noticed an egg-shaped, painless mass in the left pre-auricular region while washing his face. There were no accompanying symptoms of facial weakness, dysphagia, fever, or recent unintended weight loss. Six months later, the mass remained unchanged. Ultrasound examination at a local hospital prompted fine-needle aspiration cytology (FNAC), which was interpreted as pleomorphic adenoma. Upon admission (August 2023), physical examination revealed a 2 × 3 cm mobile, rubbery, non-tender mass over the left angle of mandible. Facial nerve function was intact (House-Brackmann grade I). No cervical lymphadenopathy was palpable. The remainder of the Otorhinolaryngology and systemic examination was unremarkable. Contrast-enhanced computed tomography (CT) at the local hospital demonstrated a well-circumscribed, mildly enhancing 2.5 cm nodule within the superficial lobe of the left parotid gland, without infiltration of the deep lobe or surrounding adipose tissue. Subsequent magnetic resonance imaging (MRI) confirmed a nodular lesion in the left parotid gland, suggestive of a neoplastic lesion, most likely a pleomorphic adenoma (**Figures 1A, B**). Chest radiography was unremarkable. Under general anesthesia, a modified Blair incision was performed. Intra-operative facial nerve monitoring was utilized. The tumor was completely excised with a 0.5 cm margin of normal parotid tissue (superficial parotidectomy), with no intra-operative tumor spillage. Intraoperative frozen section favored a low-grade malignant salivary gland tumor. Considering the potential risks of the tumor, a functional supra-omohyoid neck dissection (levels I–III) was performed, as frozen section analysis of a sentinel node—identified by peri-tumoral methylene blue injection—revealed reactive hyperplasia without metastatic involvement.

**Figure 1:**
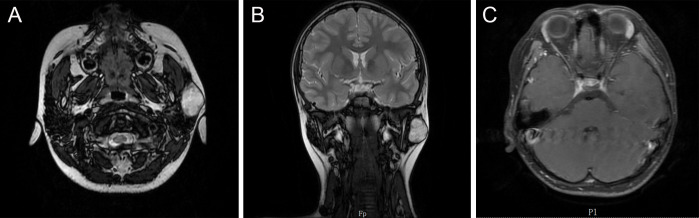
Maxillofacial MRI findings. **(A, B)** axial and coronal plane MRI of the maxillofacial region: A nodular lesion with isointense T1 and slightly prolonged T2 signals was detected in the left parotid gland, showing heterogeneous hyperintensity on fat-suppressed sequences. The lesion had relatively clear boundaries, irregular morphology, and measured approximately 1.9×2.3×2.5 cm. No obvious abnormal signals were observed in the right parotid gland or bilateral submandibular glands. **(C)** postoperative maxillofacial MRI: contrast-enhanced scan revealed postoperative changes in the left parotid gland, soft tissue edema at the surgical site, and no significantly enhancing mass.

Grossly, the specimen consisted of a 5.5 × 4 × 2 cm segment of parotid gland tissue harboring a 2.5 cm tan, partially encapsulated tumor. Histological examination revealed the following features. At low magnification, the tumor demonstrated relatively circumscribed borders with an overall nodular or microcystic architecture (**Figure 2A**). The tumor at the visible margin showed infiltrative growth in small clusters. Follicular or microcystic patterns predominated throughout the tumor, forming cystic cavities of varying sizes (**Figure 2B**). These cystic cavities contained eosinophilic secretions, and densely packed cellular arrangements were observed lining the periphery of some lumina. Tumor cells with solid sheet-like growth accounted for more than 30% of the total tumor area, where eosinophilic secretions were also evident along with focal cellular aggregates (**Figure 2C**). Under high magnification, the tumor cells exhibited relatively uniform morphology; the solid areas were predominantly composed of round, oval, or epithelioid cells with abundant eosinophilic granular cytoplasm. The nuclei were round to oval, with distinct nuclear membranes, prominent nucleoli, and a vesicular chromatin pattern. Tumor giant cells and pathological mitotic figures were readily identifiable (**Figure 2D**). Vascular invasion and perineural invasion were absent. Surgical resection margins were negative (>1 mm).

**Figure 2:**
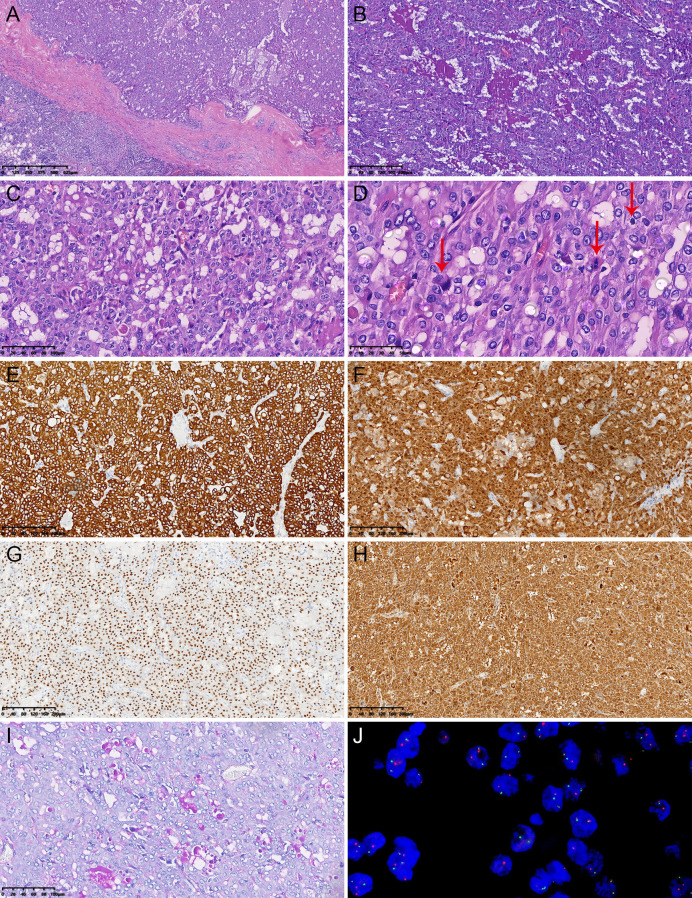
Clinical and pathological features of secretory carcinoma of the parotid gland. **(A)** at low magnification, the tumor demonstrated relatively circumscribed borders with an overall nodular or microcystic architecture. Scale bar, 625 μm. **(B)** follicular or microcystic patterns were predominant throughout the tumor, forming variably sized cystic cavities. Scale bar, 200 μm. **(C)** solid and microcystic/tubular areas with eosinophilic secretions Scale bar, 100 μm. **(D)** at high magnification, the tumor cells demonstrate relatively uniform morphology, with solid areas predominantly composed of round, oval, or epithelioid cells exhibiting abundant eosinophilic granular cytoplasm. The nuclei are round to oval, featuring distinct nuclear membranes and prominent nucleoli, with a vesicular chromatin pattern. Tumor giant cells and mitotic figures are evident (red arrow). Scale bar, 50 μm. **(E)** diffuse membranous positivity for CK19. Scale bar, 200 μm. **(F)** diffuse cytoplasmic/membranous positivity for S100. Scale bar, 200 μm. **(G)** diffuse nuclear positivity for SOX10. Scale bar, 200 μm. **(H)** diffuse cytoplasmic/membranous positivity for mammaglobin. Scale bar, 200 μm. **(I)** eosinophilic PAS-positive material localized in the cytoplasm. Scale bar, 200 μm. **(J)** FISH demonstrating *ETV6*::*NTRK3* gene fusion.

Immunohistochemical analysis demonstrated diffuse positivity for CK19 (**Figure 2E**), S-100 (**Figure 2F**), SOX10 (**Figure 2G**), and mammaglobin (**Figure 2H**); Ki67 proliferation index reached 15% in hotspot areas. Periodic acid-Schiff (PAS) staining highlighted intraluminal secretory material within the lumina (**Figure 2I**). Fluorescence *in situ* hybridization (FISH) was performed on formalin-fixed paraffin-embedded sections using a dual-fusion probe strategy targeting the *ETV6* and *NTRK3* loci (*ETV6::NTRK3* Fusion Probe Kit, HealthCare Biotechnology, Wuhan, China). An *ETV6::NTRK3* fusion was detected in 76/200 nuclei (38%; positivity threshold, 10%; **Figure 2J**). All six dissected lymph nodes exhibited only reactive changes. The final diagnosis of this case was secretory carcinoma of the parotid gland, grade 2.

The patient experienced an uneventful postoperative recovery; transient paralysis of the marginal mandibular branch of the facial nerve resolved within three weeks. Postoperative maxillofacial MRI performed two weeks later revealed postoperative changes in the parotid gland with soft tissue edema in the surgical area (**Figure 1C**). A multidisciplinary tumor board review, noting the absence of adverse pathological features (age >10 years, tumor size >1.5 cm, but absence of nodal disease or extraparenchymal extension), resulted in the decision to omit adjuvant radiotherapy or *NTRK* inhibitor therapy.

At 29 months of follow-up, the patient remained disease-free with no evidence of recurrence, preserved facial nerve function, and no radiological signs of residual or recurrent disease.

## Discussion

SC primarily occurs in adults, with a mean age at diagnosis of 42.6 years (ranging from 4 to 87 years) and a slight male predominance (6–8). However, its incidence is exceedingly rare in the pediatric population. A systematic review encompassed pediatric patients aged 10 to 17 years (median age: 15 years), where the most prevalent clinical manifestation was a slowly enlarging, fixed, painless mass, often detected incidentally during routine physical examinations (9). In a pediatric cohort from 2010 to 2023, parotid gland involvement was observed in 77.1% of cases. Notably, the incidence rates of regional and distant metastasis were calculated as 14.6% and 8.4%, respectively (10). Furthermore, higher-grade components have been documented in adult populations; however, such cases remain infrequently documented in pediatric patients. The preoperative clinical manifestations, imaging examinations, and FNAC findings in this pediatric case were consistent with a benign tumor, most likely a pleomorphic adenoma. Intraoperative frozen section pathology initially suggested a low-grade salivary gland-type tumor, prompting regional lymph node dissection and thereby obviating the need for a secondary surgical procedure. Definitive diagnosis was established based on positive immunohistochemical staining for S100, SOX10, and mammaglobin, coupled with the detection of the *ETV6::NTRK3* gene fusion. This case exhibits a high susceptibility to misdiagnosis as a benign tumor. Alena Skalova first proposed the grading criteria for SC, including tumor size, histological growth pattern, tumor fibrous septa/fibrosis/stromal hyalinization, perineural invasion (PNI), lymphovascular invasion (LVI), tumor necrosis, nuclear pleomorphism, nucleolar morphology, and mitotic activity/Ki-67 labeling index. Based on the presence, extent, or severity, each criterion was assigned a score of 1–3 points. Using the proposed scoring system, all tumors were classified into three grades: grade 1 (4–6 points), grade 2 (7–9 points), and grade 3 (10–12 points). Patients with high-grade component exhibited a significantly higher risk of recurrence and mortality compared to those without (11). Therefore, identifying high-grade components in SC is crucial. In this case, pathological morphological assessment revealed high-grade components characterized by solid growth patterns and high-grade nuclear features, including prominent nucleoli and mitotic figures exceeding 3 per 10 high-power fields (HPF). Ki67 proliferation index reached 15% in hotspot areas. Application of the proposed 12-point scoring system yielded 8 points, consistent with grade 2 secretory carcinoma with high-grade morphological features. This pediatric SC case, demonstrating high-grade morphological features, presented comprehensive diagnostic and therapeutic data encompassing preoperative clinical presentation, imaging findings, FNAC results, intraoperative records, postoperative pathological diagnosis, and follow-up information, thereby offering a valuable reference for clinical practice.

SC presents diagnostic challenges in imaging due to its nonspecific radiological features, which overlap with those of various other salivary gland tumors. On CT, SC typically manifests as a well-defined, elliptical or lobulated mass with hypodensity on unenhanced scans. Following contrast administration, it demonstrates marked heterogeneous enhancement—a pattern shared by certain benign neoplasms. MRI typically reveals SC as lesions with low or isointense signal intensity on T1-weighted images and high or mixed signal intensity on T2-weighted images, accompanied by heterogeneous post-contrast enhancement (12). These imaging similarities necessitate that definitive diagnosis integrates clinical presentation, histopathological examination, and molecular data.

FNAC represents a guideline-recommended standard diagnostic modality for the preoperative assessment of salivary gland tumors in adults. However, its diagnostic efficacy in the pediatric population remains controversial, primarily due to the paucity of robust data and inherent challenges, including the requirement for patient cooperation, potential requirement for sedation, and associated procedural risks (13). In children, the clinical impression of a benign lesion is further heightened by the rarity of SC and its indolent growth pattern. The case presented herein was initially erroneously diagnosed as a pleomorphic adenoma. Consequently, definitive pathological examination and molecular work-up are indispensable. The pathological features of SC typically include well-circumscribed nodular or microcystic tumors. Microscopically, the tumor cells are of moderate size with small, pale nuclei and eosinophilic cytoplasm containing granules or vacuolar structures, arranged in microcystic, tubular, or solid patterns. The nuclei are round to oval, featuring distinct nuclear membranes and prominent nucleoli, with a vesicular chromatin pattern. Tumor giant cells and pathological mitotic figures are evident. Ki67 proliferation index reached 15% in hotspot areas. The overall rating climbed to an impressive 8 points. These all confirm the existence of high-grade morphology. The luminal spaces contain eosinophilic secretions that are PAS-positive. Immunohistochemically, SC is positive for S-100, SOX10, CK19, and mammaglobin, while negative for myoepithelial markers and DOG1. The differential diagnosis includes pleomorphic adenoma, mucoepidermoid carcinoma, acinic cell carcinoma, and low-grade mucoepidermoid carcinoma. Pleomorphic adenoma is characterized by mixed epithelial and mesenchymal components; it is immunohistochemically positive for S-100 and SOX10 but weakly expressive of CK19 and mammaglobin and lacks the *ETV6::NTRK3* fusion. Mucoepidermoid carcinoma is composed of mucinous cells, epidermoid cells, and intermediate cells; it is immunohistochemically positive for CK7 and CK19 but weakly expressive of S-100 and SOX10 and lacks the *ETV6::NTRK3* fusion. Adenoid cystic carcinoma exhibits cribriform and tubular patterns; it is immunohistochemically positive for CK7, CK19, and S-100 but weakly expressive of SOX10 and mammaglobin and lacks the *ETV6::NTRK3* fusion. Low-grade polymorphous carcinoma displays diverse cellular morphology; it is immunohistochemically positive for CK7 and CK19 but weakly expressive of S-100 and SOX10 and lacks the *ETV6::NTRK3* fusion (8, 14).

The detection of the *ETV6::NTRK3* gene fusion is pathognomonic for SC. The World Health Organization (WHO) 5th Edition Classification of Head and Neck Tumors recommends the routine identification of this fusion using fluorescence *in situ* hybridization (FISH) or next-generation sequencing (NGS) (15). A 2020 molecular study of salivary gland SC found that among 49 samples, 40 (82%) harbored the classic *ETV6::NTRK3* fusion, while 9 (18%) exhibited alternative fusions. Of these 9 alternative cases, 8 carried the *ETV6*::*RET* fusion, and 1 parotid tumor harbored a novel *VIM*::*RET* fusion identified via NGS. Additionally, one recurrent submandibular tumor co-harbored *ETV6*::*NTRK3* and *MYB*::*SMR3B* fusions (16). Therefore, further collection of cases is required to comprehensively elucidate the molecular alterations of SC.

A meta-analysis of 256 patients with parotid gland SC demonstrated that 76.3% presented with T1 or T2 stage tumors and negative cervical lymph node status. Among patients with T4 stage tumors, the incidence rates of lymph node metastasis (57.4%) and distant recurrence (46.2%) were significantly higher (17). Although SC is currently classified as a low-grade malignancy with a comparatively favorable prognosis, it retains the potential for aggressive biological behavior. Case reports have documented adult patients presenting with local recurrence, high-grade transformation, as well as regional lymph node or distant metastases. Such patients often require postoperative adjuvant radiotherapy, chemotherapy, and occasionally targeted therapy (9). Tumor grade is closely associated with the risk of distant metastasis. It was reported that the patients with grade 2 or 3 tumors have a significantly shorter time to distant metastasis compared to those with grade 1 tumors (11). Yokota et al. reported a multicenter study in which four patients with advanced SC harboring the *ETV6*::*NTRK3* gene fusion achieved durable radiographic complete responses (CR) following entrectinib treatment, with three patients remaining on therapy at the time of reporting (18). However, concerning treatment strategies, clinical outcomes, and prognostic data for pediatric SC, evidence remains limited to case reports or small-sample studies. Consequently, comprehensive long-term data collection and follow-up observation are critically required. Current management of pediatric SC primarily centers on complete surgical resection. For patients exhibiting high-risk features (e.g., high-grade morphology, positive surgical margins, perineural invasion, or lymphovascular invasion), standardized adjuvant therapy protocols are lacking. Cardoni et al. recently described a pediatric case of high-grade SC arising in the maxillary sinus, suggesting that tumor necrosis and elevated mitotic activity within the tumor may portend a more aggressive clinical course (14). Notably, however, the patient in that study who underwent complete resection demonstrated no recurrence during follow-up exceeding 4 years. Despite the presence of characteristic high-grade components in the present case, a modified Blair incision was adopted for surgical resection. Intraoperative facial nerve monitoring was applied, and the tumor was completely excised with a 0.5 cm margin of normal parotid tissue preserved—this approach ensured tumor integrity and prevented intraoperative rupture. Postoperative pathological examination showed no definitive evidence of vascular tumor thrombi or perineural invasion.

A multidisciplinary team (MDT) discussion was carefully conducted, leading to a consensus on the treatment strategy: exclusive surgical intervention followed by rigorous postoperative surveillance. This case demonstrates that even in pediatric SC with high-grade components, a favorable prognosis can be achieved through meticulous surgical techniques and strict postoperative follow-up management. Furthermore, this case serves as a crucial reminder to clinicians: when managing pediatric parotid neoplasms, even if preoperative evaluations strongly suggest a benign lesion, heightened vigilance is imperative. Where clinically appropriate, more comprehensive pathological examinations and molecular testing should be proactively pursued to ensure accurate diagnosis and optimal patient outcomes.

## Data Availability

The original contributions presented in the study are included in the article/supplementary material. Further inquiries can be directed to the corresponding author.
